# 

**Published:** 1889-01

**Authors:** 


### INDEX TO VOLUME XLIII.

### COMMUNICATIONS.

A Brief Description of Shedding Temporary Teeth, as to Time, Order, and Process 12
Address 577
A Study of the Chemical Composition of the Dental Pulp. 581
Aluminum 17
Association 170
A Criticism 230
A Brief Treatise on the Common Diseases of the Maxillary Sinus 265
A Review by J. C. Reeve, M.D, of Dayton, Ohio, on Anæsthetics in Dental Practice. 21
Characteristics of Alloys 112
Calcific Inflammation 167
Copper Amalgam 282
Cocaine Anæsthesia 323
Clinical Instructions 540
Dental Transplantation and Replantation 57
Diseases of Mucous Membranes. 476
Excelsior 595
Etiology of Caries 390
Fractured Jaw 439
Facial Neuralgia Consequent
Failures 592
upon Pregnancy 473
Filling Root Canals of Human Teeth 333
Mistakes 172
Name and Describe the Causes of Dental Caries 213
One Way of Increasing a Dentist's Usefulness 589
President's Address, by H. L. Moore, D.D.S 161
President' Address—Prof. C. M. Wright, D.D.S 577
Pulp Stones 232
President's Address—Meeting of M. D. A., Grand Rapids. 317
Report of Seventh District Ohio Dental Society 597
Restoring Articulation of Tipping Molars with Gold 347
Reflex Neurosis in Relation to Dental Pathology 398
Shedding Temporary Teeth as to the Process 65
Short Notes on Diseases of the Palate 178
Scurvy 233
Treatment of Temporary Teeth. 5
The Third Molar 8
The Physician and Dentist 69
The Chicago Dental Society 117
The Nervous Patient 164
The Use of the Electro-Magnetic Mallet 174
The Peridental Membrane 228
The Survival of the Unfit in Human Dentition 345
The President's Address 370
The Relation of the Teeth to the Diseases of the General System 421
The Third Molar, Wisdom Tooth, or Dens Sapientiæ 430
The Influence of the Various Deposits upon the Teeth and Adjacent Parts 482
The Co-Relation of General and Dental Medicine and Surgery 525
The Dentist's Right to Practice Medicine 536
The Disputes between the Editors of the Dental Journals. 599
What Varieties in Number are Found in the Teeth of Mammals? 10
What Variations in the Manner of Using the Teeth are Exhibited by Various Animals Including Man 15
When is Extraction a Necessity 110
What Variations in the Manner of Using the Teeth are Exhibited by the Various Animals Including Man 221
What Shall We Do in the Case? 339
Woman's Work in the Profession 586

PROCEEDINGS.

Georgia State Dental Society.... 504
National Association of Dental Faculties .................... 512
National Association of Dental Examiners...................... 515
Fifth Annual Meeting of the Ohio State Dental Society... 601
Proceedings of the American Dental Association............ 485
The Odontological Society of Pennsylvania.................. 100
The Iowa State Dental Society. 442
By-Laws of the Dental Protective Association of the U. S. 44
Chicago Dental Society Meetings 48
To Members of the Dental Profession........................... 42
A Bill for an Act to Regulate the Practice of Dentistry in the State of Minnesota...... 303
Monthly Digest for May.......... 223
Operative Dentistry............... 149
The Trend of Dental Thought as Presented by our Contemporaries. 31, 75, 90, 182, 292

SELECTIONS.

Absorption of the Tissues of the Mouth from Pressure......... 197
American Medial Association... 313
A New Organization—A District Society......................... 314
A Simple Inhaler..................... 414
Abortive Treatment of Whitlow. 465
Anæsthesia ............................ 616
A Strange Use of Hypnotism.... 559
An Extraordinary Advertisem't 568
Anæsthetics and Accidents...... 613
Brain-Workers........................ 258
Bacteria in Milk..................... 358
Bacteriology and Practice........ 550
Babies Beginning to Walk....... 563
Chloroform as an Anæsthetic.... 615
Cocaine in Teeth Extraction..... 364
Capping Exposed Pulps........... 357
Diseases in Adults and in Children.................................. 147
Deaths under Anæsthetics........ 609
Dental Fistulæ....................... 357
Effects of Mental Overwork...... 253
Early Rising........................... 567
Hare-Lip and Cleft Palate........ 464
He that Bloweth not his own Horn the Same will not be Tooted.............................. 557
Influence of Certain Medical Agents on the Bacillus of Tubercle in Man.............. 205
International Dental Congress. 359
The International Medical Congress of 1890..................... 621
Ideal for a Medical Society...... 362
Ice-Water ............................. 462
Method of Making Sections of Teeth and Preserving the Delicate Parts.................. 196
Methods of Instruction in Medicine................................ 353
Microzoa ............................... 355
Microscopical Laboratory Notes 562
Micro-Organisms of Dental Caries.............................. 569
New Appliances..................... 313
Notes of Surgical Diseases of Children........................... 561
Obituary .............................. 315
Odontological Society of Great Britain ........................... 350
Officers of the American Medical Association................. 570
Pea-Soup as a Substitute for Beef-Tea ......................... 619
Physical Education................. 459
Phenacetin in the Treatment of Neuralgia ....................... 463
Preparing and Mounting Sections of Brain.................. 619
Prof. Mendel's Views of the Therapeutic Value of Hypnotism.............................. 519
Quinine and Tannic Acid......... 415
Rules for Good Health............ 566
Skin-Grafting ......................... 150
States May Regulate Medical Practice........................... 257
State Registration of Trained Nurses........................... 560
The Air of the Bed-Room......... 148
The Nail Brush Question.........
To Preserve Blood Corpuscles for Microscopic Examination.................................. 197
Therapeutics of Iodol............. 198
The Tooth in the Apple........... 199
To the Editor of the Dental Register........................... 200
Transactions of the Ninth International Medical Congress. 259
The Alcoholic and Opium Habit 413
The Medical Student of the Future............................ 553
What Kills the Children......... 253

DEN. COL. COMMENCEMENTS.

Baltimore College of Dental Surgery .... 245
Columbia University—Dental Department .... 247
Central Tenn. College School of Dentistry .... 248
Howard University — Dental Department .... 248
Indiana Dental College .... 243
Kansas City Dental College .... 248
Missouri Dental College .... 246
New York College of Dentistry. 250
Ohio College of Dental Surgery. 244
Philadelphia Dental College .... 249
Pennsylvania College of Dental Surgery .... 252
Royal College of Dental Surgeons of Ontario .... 250
St. Louis College of Physicians and Surgeons—Dental Department .... 249
Southern Medical College—Dental Department .... 247
University of Tennessee—Dental Department .... 252
Vanderbilt University — Department of Dentistry .... 251

EDITORIALS.

A Change .... 55
Annual Meeting of the Mississippi Valley Association of Dental Surgeons .... 106
A New Text Book .... 107
American Medical Association. 209
Alumni Notice .... 263
American Medical Association. 313
A New Organization—A District Society .... 314
American Medical Association. 364
American Dental Association ... 364
American Dental Association ... 367
American Dental Association ... 466
A Crown and B idge Case, 1859. 469
An Interesting Case .... 416
A Suggestive Case .... 417
Biographical .... 158
Bibliographical .... 210
Biographical .... 210
Buried Gold .... 523
Board of Dental Examiners Meeting .... 524
Bibliographical Aide-Memoire of the Surgeon Dentist .... 571
Close of the Volume .... 622
Dentist Appointment Book .... 56
Dental Department of Meharry Medical College .... 107
Dental Protective Association... 153
Dental Society Meetings .... 260
Dr. Wm. H. Dwinelle .... 468
Dr. Berry's Death .... 575
Educational .... 154
Explanatory .... 419
Fifth Annual Meeting .... 523
For Sale .... 524
Hayden Dental Society of Chicago .... 471
Libraries .... 206
Literary Items .... 210
Meeting of the Mississippi Valley Dental Society .... 105
Medical and Dental Department Central University of Ky... 470
New Appliances .... 313
Obituary .... 55
Ohio District Dental Societies... 208
Obituary .... 315
Obituary .... 368
Ohio Valley Dental Society .... 522
Ohio State Society .... 522
Programme St. Louis Dental Society .... 157
Pre-historic Dentistry—Bridge Work .... 261
Post-Graduate School .... 264
Painless Destruction of Naevix. 420
Personal .... 575
Special Notice .... 261
Society Meeting—Seventh District Ohio .... 471
The National Association of Dental Examiners .... 366
The National Association of Dental Faculties .... 366
To Saratoga .... 367
The First Matches .... 367
The Association of Dental Faculties .... 467
Transactions .... 471
The Dental Protective Association of the United States... 520
The Seventh Ohio District Dental Society .... 521
The Post Graduate School of Prosthetic Dentistry .... 573
Waste no Time .... 160

### DIRECTORY OF DENTAL SOCIETIES.

*American Dental Association*—Meets on the first Tuesday of August, 1889 at Saratoga. Pres. C. R. Butler, Cleveland, O.; Rec. Sec. Geo. H. Cushing, Chicago.

*Southern Dental Association*—Meets in Galveston, Texas. President J. Y. Crawford, Nashville, Tenn., Secretary M. C. Marshall, Little Rock, Ark.

### STATE DENTAL SOCIETIES.

*Alabama Dental Association*—Meets annually. Next meeting at Mobile, on the second Tuesday of April, 1889. Pres. A. Ewbank, Birmingham; Sec. R. Y. Jones, Florence.

*California State Dental Association*—Third Tuesday in July, 1889. Pres. W. De Crow, San Josie; Rec. Sec. W. A. Knowles, San Francisco; Cor. Sec. W. Z. King, San Francisco. Next Meeting in San Francisco.

*Colorado State Dental Society*—Pres. J. W. Grannis, Sec. M. P. Ke[illegible]og, Denver; Next meeting will be held in Denver, June 5th, 1889.

*Connecticut State Dental Society*—Pres. E. A. Stebbins, Shelburne Falls; Rec. Sec. Geo. A. Maxfield, Holyoke. Annual meeting Oct. 27th and 28th, 1887, at Hartford.

*Dental Society of the State of Maryland and District of Columbia*—Meets annually. Next meeting at Washington City, on the second Tuesday in October, 1888. Pres. B. F. Coy, of Baltimore: Sec. M. W. Foster, M. D.

*Georgia State Dental Society*—Meets second Tuesday in May, 1889, at Tybee; Pres S. A. White: Rec. Sec. H. H. Johnson; Cor. Sec. L. D. Carpenter, Atlanta.

*Illinois State Dental Society*—Meets annually. Pres. Geo. H. Cushing, Chicago; Sec. G. Newkirk, Chicago. Next place of meeting, Quincy, second Tuesday in May, 1889.

*Indiana State Dental Society*—Meets next in Indianapolis on the last Tuesday of June, 1889. Pres. J. B. Morrison, Indianapolis; Sec. Robert W. Van Valzah, Terre Haute.

*Iowa State Dental Society*—Meets annually. Next meeting in Des Moines, on the first Tuesday in May, 1889. Pres. J. B. Montfort, Fairfield; Rec. Sec. G. W. Miller, Des Moines.

*Kentucky State Dental Association*—Meets annually, first Tuesday in June, 1889. Next meeting in Louisville. Pres. J. M. Baldwin, Sec. C. E. Dunn, Louisville.

*Kansas State Dental Association*—Pres. W. M. Shirley, Hiawatha; Sec. C. B. Gunn, Leavenworth. Next meeting will be held at Topeka, April 30th, 1889.

*Maine Dental Society*—Meets in August, at Thomaston. Pres. E. J. Robert, Augusta, Sec. C. E. Hussey, Beddeford.

*Maryland State Dental Association*—Meets annually in February. Pres. Wm. A. Mills; Rec. Sec. Chas. F. Dingler; Cor, Sec. S. C. Pennington

*Massachusetts Dental Society*—Meets annually in Boston, second Thursday in December. Pres. S. G. Stevens, Boston; Rec. and Cor. Sec. G. F. Eames, Boston.

*Michigan State Dental Association*—Meets annually. Next meeting at Grand Rapids, Tuesday, May, 7th, 1889. Pres. C. S. Case, Jackson; Sec. Wm. Cleland, Detroit; Treas. H. K. Lathrop, Detroit.

*Mississippi State Dental Association*—Will meet third Tuesday in May, 1889, at Vicksburg. Pres. W. H. Marshall, Oxford; Sec. A. H. Hilzim, Jackson.

*Minnesota Dental Association*—Meets annually second Wednesday in July, 1889, at St. Paul. Pres. H. L. Cruttenden, Northfield; Sec D. W. Edwards, Le Sueur.

*Missouri Dental Association*—Meets annually on July 10th, 1888, Next meeting at Pertel Springs. Pres. Wm. N. Morrison, St. Louis; Sec. John G. Harper, St. Louis.

*Nebraska State Dental Society*—Meets annually. Next meeting third Tuesday in May 1888, at Beatrice; Pres. J. J. Willey, Wahoo; Sec. C. R. Tefft, Lincoln.

*New Jersey State Dental Society*—Meets annually. Next meeting at Asbury Park, on the third Wednesday in July, 1889. Pres. A. Hull, New Brunswick; Sec. C. A. Meeker, Newark.

*New Hampshire Dental Society*—Meets in Concord on the third Tuesday of June, 1889. Pres. C. H. Maynard, Peterboro; Sec. E. B. Davis, Concord.

*North Carolina State Dental Society*—Meets annually. Next meeting at Ashville; fourth Tuesday in July, 1889. Pres. V. E. Turner, Raleigh; Sec. H. C. Hearing, Concord.

*Ohio State Dental Society*—Meets annually. Next meeting at Cleveland, last Tuesday of October, 1889. Pres. C. M. Wright, Cincinnati; Sec. J. R. Callahan, Hillsboro.

*Pennsylvania State Dental Society*—Meets second Tuesday of September, 1888, at Philadelphia; Pres. W. H. Roop Philadelphia; Rec. and Cor. Sec. Th. F. Chupein, Philadelphia.

*Rhode Island Dental Association*—Pres. A. W. Buckland, Woonsocket; Sec. W. P. Church, Providence. Meets quarterly, first Tuesday in July, October, January, and April. Annual Meeting first Tuesday in July, 1888.

*South Carolina State Dental Association*—Meets annually. Pres. L. S. Wolfe, Orangburg; Cor. Sec. E. C. Ridgell, Batesburg; Rec. Sec. R. A. Smith, Charleston. Next meeting at Greenville, third Tuesday in July, 1888.

*Texas State Dental Association*—Meets in Buton, May 1st, 1888. Pres. W. J. Barton, Paris; Sec. G. M. Patten, Huntsville.

*Tennessee Dental Association*—Meets Annually. Next meeting at Memphis, first Tuesday in July 1888. Pres. Gordon White, Nashville; Rec. Sec. D. R. Stubelfield, Nashville; Cor. Sec. H. E. Beach, Clarksville.

*The Dental Society of the State of New York*—Meets annually on the second Wednesday in May. Next session at Albany, May 8, 1889. Pres. J. Edw. Line, Rochester; Rec. Sec. M. S. Jewell, Richfield Spa.; Cor. Sec. G. L. Curtis, Syracuse.

*Vermont State Dental Society*—Meets annually. Next meeting at Montpelier, March 20, 1889. Pres. R. W. Warner, St. Johnsbury; Sec. Thos. Mound, Rutland.

*Virginia State Dental Association*—Meets in Charlottsville, Tuesday, August 19, 1888. Pres. J. Hall Moore; Sec. L. M. Cowarden, Richmond.

*Wisconsin State Dental Society*—Meets annually. Next meeting at Milwaukee, July 20, 1888. Pres. B. G. Marklein, Milwaukee; Sec. C. A. Southwell, Appleton, Wis.

### LOCAL SOCIETIES.

*American Academy of Dental Science*—Meets annually in Boston, Mass., on the first Wednesday in October, 1888. Pres. J. H. Batchelder; Rec. Sec. E. E. Hopkins; Cor. Sec. E. B. Hitchcock, Boston.

*Brooklyn Dental Society and Second District Society United*—Meets quarterly—Pres. T. H. Holly; Rec. Sec. A. N. Russell, Brooklyn.

*Central Dental Association of Northern New Jersey*—Meets annually in February. Pres. C. A. Meeker, Newark, Sec. J. G. Palmer, New Brunswick.

*Central Illinois Dental Society*—Meets annually. Next meeting at Lincoln, Oct. Oct. 9 and 10, 1888; Pres. J. M. Fishburn, El Paso; Sec. W. A. Johnston, Peoria.

*Chicago Dental Society*—Meets monthly. Pres. F. H. Gardiner; Sec. J. G. Reid.

*Chicago Dental Club*—Meets on the fourth Monday of each month. Pres. A. B. Freeman; Sec. C. Stoddard Smith.

*Cleveland Dental Society.*—Meets the first Monday of each month. Pres. Ira Brown; Sec. Jere E. Robinson.

*Connecticut Valley Dental Society*—Meets 27 and 28 of Oct. 1888, at Savin Rock Conn. Pres. C. T. Stockwell, Springfield, Mass. Sec. A. M. Ross, Chicopee.

*Detroit Dental Society*—Meets monthly, Pres. Geo. S. Shattuck.

*Eighth District Dental Society, N. Y.*—Meets semi-annually. Next meetlng in Syracuse, Oct. 24-26, 1888. Pres. T. E. Howard; Sec. S. A. Freeman, Buffalo.

*East Tennessee Dental Association*—Meets annually. Next meeting will be held at Chattanooga, Tenn., on the first Tuesday of Aug., 1888. Pres. R. A. B. Noyers, Sec. S. N. Prothro.

*Mad River Dental Society*—Meets annually. Next meeting at Dayton, Tuesday, May 19, 1889. Pres. C. Bradley, Dayton; Sec. and Treas. L. C. Adams, Dayton,

*Mississippi Valley Dental Society*—Meets annually at Cincinnati, on first Wednesday in March, 1889. Pres. H. L. Moore, Cincinnati, Ill.; Sec. A. G. Rose, Cincinnati.

*New England Dental Society*—Meets annually. Time and place of next meeting in Boston, November 15th, 1888. Pres., A. M. Dudley, Salem, Mass.; Sec. A. H. Gilson, Boston, Mass.

*Northern Illinois Dental Society.*—Meets annually. Next meeting at Freeport, October —, 1889. Pres. J. H. Cormany, Mt. Carroll. Sec. T. W. Beckwith.

*Northern Ohio Dental Association*—Meets annually. Next meeting at Cleveland, O. on the second Tuesday in May, 1889. Pres. H. F. Harvey, Cleveland, O.; Rec. Sec. W. H. Whitsler, Youngstown; Cor. Sec. S. B. Dewey, Cleveland, O.

*Northwestern Dental Association* (embraces portions of Dakota, Minnesota and Manitoba) meets annually. Next meeting at Detroit, Dak. on the fourth Tuesday in July, 1889. Pres. J. W. Cloes, Jamestown, Dak. Sec. S. J. Hill, Fargo, Dak.

*Odontological Society of Pennsylvania*—Meets on the first Saturday evening of each month, at 8 o'clock p. m., in the offices of the members; Pres. E. T. Darby, Rec. Sec. Ambler Tees.

*Odontological Society of New York City*—Meets the third Tuesday of each month. Pres. Wm. Jarvie; Rec. Sec. E. T. Payne.

*Odontological Society of Cincinnati*—Meets monthly. Pres. J. S. Cassidy, Sec. M. H. Fletcher.

*Pennsylvania Association of Dental Surgeons*—Meets second Tuesday of each month; Pres. H. E. Roberts; Rec. Sec. Theo. F. Chupein.

*Pittsburg Dental Society*—Meets the last Tuesday Evening of each month. Pres. J. B. Williams, Homestead; Sec. N. L. Reinecke.

*Fifth District Dental Society of N. Y.*—Meets semi-annually. Next meeting in Syracuse, October, 24-26. Pres. R. F. Jones, Utica; Sec. T. B. Mason, Phoenix.

*San Francisco Dental Society*—Meets the second Monday evening of each month. Pres. W. A. Knowles; Sec. Chas. Morffew; Treas. J. J. Birge.

*Sixth District Dental Society of N. Y.*—Meets semi-annually. Next meeting at Syracuse, Oct. 24-26, 1888. Pres. E. D. Downs, Owego; Sec. Myron D. Jewell, Richfield Springs.

*St. Louis Dental Society*—Meets first Tuesday in each month. Pres. Henry Fisher; Cor. Sec. Wm. Conrad; Rec. Sec. John H. Spalding.

*Washington Dental Society*—Meets second Tuesday of each month. Pres. R. Finley Hunt; Rec. Sec. H. M. Schooly.

*First District Dental Society of the State of New York*—Meets annually. Pres. A. L. Northrop; Sec. J. E. Dexter, New York.

*Washington City Dental Society*—Pres. M. B. Noble; Sec. Wm. Donally. Meets monthly.

*The Western Illinois District Dental Association*—Meets at Kewanee, October 16, Pres. F. Christianer, Abington; Sec. R. W. Bailey, Macomb.

---

### EXCHANGES

St. Louis, Medical and Surgical Journal.
The Pacific Medical and Surgical Journal, San Francisco.
Cincinnati Lancet and Clinic.
Dental Cosmos, Philadelphia.
Chicago Medical Examiner.
The Detroit Review of Medicine and Pharmacy.
American Journal of Dental Science, Baltimore.
Archives of Dentistry.
L'art Dentaire, Paris.
Le Progres Dentare, Paris.
Deutsche Monatsschrift fuer Zahnheilkunde Leipzig.—Arthur Felix.
Ohio State Journal of Dental Science, Xenia, Ohio
Independent Practitioner, N. Y.
The Dental Record, London.
The Journal of the British Dental Association, London.
The Southern Dental Journal.
Items of Interest, Philadelphia.
The Dental Practitioner.—The Cincinnati "Medical" News.
The British Journal of Dental Science, London, Eng.
The Dental Advertiser.
The Messenger of Dentistry, Russian.
The Medical World.
The American Pharmacist.
The Nashville Journal of Medicine and Surgery.
Medical and Surgical Reporter, Philadelphia, Pa.
Dental Headlight.
Cincinnati Medical and Dental Journal.
The New York Medical and Surgical Journal.
The Dental Review.

### Chicago Dental Society Meeting.

The twenty-fifth anniversary of the Chicago Dental Society will be celebrated by a three day's meeting to be held in the ladies' ordinary of the Grand Pacific Hotel, corner of Clark and Jackson streets, Chicago, Ill., February 5, 6, and 7, 1889.

PROGRAMME.

TUESDAY MORNING FEB. 5, 1889.

The meeting will be called to order promptly at 10 o'clock.

PRAYER.

REV. G. C. LORIMER, D.D.......................... Chicago, Ills.

PAPER—" Gum-colored Porcelain Fillings."

A. H. THOMPSON.......................................Topeka, Kans.

DISCUSSION.

PAPER—" A Study of the Effects of Cocaine upon Man and Some of the Lower Animals."

C. P. PRUYN,..........................................Chicago, Ills.

DISCUSSION.

PAPER—"Obtundents of Sensitive Dentine."

T. E. WEEKS.......................................Minneapolis, Minn.

---

TUESDAY EVENING, 7.30 O'CLOCK.

PAPER—"The Study of Pre-historic Remains in their Relation to Dentistry."

J. J. R. PATRICK.......................................Belleville, Ills.

DISCUSSION.

PAPER—"Caries and Necrosis in their Relation to Practical Dentistry."

J. H. MARTINDALE............... .............Minneapolis, Minn.

DISCUSSION.

---

WEDNESDAY MORNING, 9 OCLOCK.

CLINICS AT THE CHICAGO COLLEGE OF DENTAL SURGERY.

---

WEDNESDAY AFTERNOON, 3 O'CLOCK.

PAPER—"Antiseptics."

G. V. BLACK....... .....................................Chicago, Ills.

DISCUSSION.

---

WEDNESDAY EVENING, 7:30 O'CLOCK.

PAPER—With Lantern Illustrations—"The Development of the Teeth, the Formation of Dentine, and its Appearance in Health and Decay."

The paper will be illustrated by photo-micrographs, projected on the screen by means of the oxy-hydrogen lantern. Many of the photographs were made for this demonstration; others are from Dr. W. D. Miller's beautiful lectures of Natural and Artificial Decay.

R. R. ANDREWS .....................................Cambridge, Mass.

DISCUSSION.

PAPER—"Artistic Methods in Prosthetic Dentistry." Illustrated by large cartoons.

L. W. COMSTOCK.............. ....................Indianapolis, Ind.

DISCUSSION.

---

CLINICS will be held on Wednesday and Thursday mornings at the Chicago College of Dental Surgery, northeast corner Wabash Avenue and Madison Street. They will begin promptly at 9 o'clock.

WEDNESDAY, FEB. 6.

J. B. VERNON........................................St. Louis, Mo.

BRIDGE WORK.

C. THOMAS........................................Des Moines, Iowa.

PORCELAIN FILLINGS.

F. PEABODY........................................Louisville, Ky.

FILLING ROOT CANALS WITH LEAD POINTS.

C. S. CASE........................................Jackson, Mich.

WILL DEMONSTRATE HIS METHOD OF MAKING ARTIFICIAL VELA AND OBTURATORS FOR CLEFT PALATE, PROVIDED A SUBJECT CAN BE SECURED.

A. H. THOMPSON........................................Topeka, Kansas.

GUM-COLORED PORCELAIN FILLINGS.

E. T. DARBY........................................Philadelphia, Pa.

FILLING WITH CRYSTAL GOLD; AND THE USE OF THE MATRICES.

A. W. HOYT........................................Chicago, Ill.

PORCELAIN FILLINGS SECURED BY GOLD FILLINGS.

S. G. PERRY........................................New York City,

WILL DEMONSTRATE THE APPLICATION OF PERRY'S SEPARATORS, AND THE WEBER-PERRY ENGINE AND MALLET.

T. E. WEEKS........................................Minneapolis, Minn.

SETTING OF LOGAN CROWN WITH GOLD ATTACHMENT, SHOWING ORIGINAL METHOD OF INVESTMENT FOR SOLDERING.

D. F. MCGRAW........................................Mankato, Minn.

OBTUNDING OF SENSITIVE DENTINE, AND CONTROLLING OF PERI-DENTAL INFLAMMATION BY ELECTROLYSIS.

J. W. WICK........................................St. Louis, Mo.

HIS METHOD OF GOLD FILLING.

LOUIS OTTOFY........................................Chicago, Ill.

IMPLANTATION.

---

THURSDAY FEB. 7.

T. D. GILMER........................................Quincy, Ill.

GOLD CROWN TELESCOPED OVER A PLATINUM BAND; ALSO A COMBINATION CROWN OF PLATINUM AND WESTON'S METAL, OR OF GOLD, PORCELAIN, AND WESTON'S METAL.

E. H. Allen........................................................Freeport, Ill.

GOLD FILLING, USING ELECTRIC MALLET.

C. N. Johnson........................................................Chicago, Ill.

GOLD FILLING.

C. W. Lewis........................................................Chicago, Ill.

HERBST METHOD.

J. G. Reid........................................................Chicago, Ill.

COPPER AMALGAM.

M. E. Smith........................................................Chicago, Ill.

GOLD FILLING, USING SNOW & LEWIS PLUGGER.

W. H. Taggart........................................................Freeport, Ill.

WILL SHOW A NEW ROOT TRIMMER, AND A NEW SUSPENSION ENGINE.

J. W. Wassall........................................................Chicago, Ill.,

WILL DEMONSTRATE ROOT FILLING WITH CHLORA-PERCHA AND GOLD POINTS ; ALSO, THE USE OF MC'KELLOP'S PLATINUM GOLD BROACHES.

E. A. Royce........................................................Chicago, Ill.

GOLD FILLING, USING ABBEY'S NON-COHESIVE GOLD IN CYLINDERS.

T. S. Waters........................................................Baltimore, Md.

MOVABLE BRIDGE.

R. B. Winder........................................................Baltimore, Md.

MOVABLE BRIDGE ; ALSO A NEW RUBBER DAM CLAMP, COMBINED WITH A CHECK HOLDER ; ALSO A SEPARATOR.

Henry A. Parr........................................................New York City.

MOVABLE BRIDGE.

J. A. Woodward........................................................Philadelphia, Pa.

REFLECTOR FOR LIGHTING THE MOUTH.

J. N. Farrar........................................................New York City,

IS EXPECTED TO BE HERE AND WILL EXHIBIT HIS REGULATING APPLIANCES.

J. N. Crouse,<br>Geo. H. Cushing, } Ex. Com.<br>E. Noyes,

The Grand Pacific Hotel will be the headquarters for guests, and will furnish rooms above the parlor floor, with board, at three dollars per day. All other rooms at fifty cents per day less than usual rates.

The Committee expect to secure railroad rates, therefore the *usual receipts should be taken* when railroad tickets are purchased showing the payment of full fare, and the Committee will secure reduced rates returning where it is possible.

An exhibit will be made by manufacturers and dealers of novelties in their various lines.

The meetings will he exclusively devoted to the reading of papers and the discussion of professional subjects and no other business will be transacted.

### EDITORIAL.

### A Change.

As will be noticed in this number of the REGISTER, that a change has been introduced, which consists in a "*monthly digest*" instead of the department of Selections, as heretofore. This will embrace the gist of the current periodical literature of the profession in as condensed form as may be consistent with clearness of statement.

The objeet is to give the meat without the bark or shell. And furthermore, most dentists take but one or two journals, and so lose whatever is of value in all others. If success attends this effort, that can not be said of the readers of the REGISTER at least.

This is not a prophesy, nor a radient promise—we leave that to those who can do it so much better; but it is a simple statement, as to a little change that is hoped may be for the better, and more interesting and profitable to the readers of the REGISTER. In the preparation of this department the valuable aid of Mrs. M. W. J. has been secured, and it is well known that whatever she attemps is sure to be well done.

### Obituary.

Died at his home in Alameda, California, Dr. C. C. Knowles, on Monday, December 17th, 1888, in his 70th year. He was a native of New Hampshire. In early life he moved to Lowell, Massachusetts, and studied dentistry with Dr. Ambrose Lawrence, a practitioner well known in his profession. Dr. Knowles

### CONTENTS.

COMMUNICATION.

Treatment of Temporary Teeth........ 5

The Third Molar........ 8

What Varieties in Number are Found in the Teeth of Mammals........10

Brief Description of the Shedding of Temporary Teeth, as to Time, Order and Process........12

Variations of Using Teeth in Various Animals........15

Aluminum........17

Anæsthetic in Dental Practice........21

MONTHLY DIGEST........31

Protection........42

By-Laws of the Dental Protective Association of the United States........45

Chicago Dental Society Meeting........48

EDITORIAL.

1889........52

1856—1889........54

A Change........55

Obituary........55

Dentists' Apportionment Book........56

---

### THE DENTAL REGISTER,

A MONTHLY MAGAZINE DEVOTED TO THE INTERESTS OF THE

**DENTAL PROFESSION.**

*Terms Reduced to* $2.00 *Per Year. Single Copies,* 25 *Cents.*

**EDITED BY PROF. J. TAFT, D.D.S.**

—AND—

**Published by SAM'L A. CROCKER & CO., Ohio Dental and Surgical Depot.**

The volume commences in January and closes in December.

The REGISTER being issued monthly is always fresh, therefore Indispensable to the practitioner who desires to keep posted up with the new inventions and improvements which are daily developed. Neither expense nor effort will be spared to give value and variety to its contents. Articles will be illustrated with well executed engravings whenever they will add to the interest or assist to explanation.

Its extensive circulation and frequency of issue make it one of the BEST DENTAL ADVERTISING MEDIUMS in the United States.

All subscriptions must begin with January or July.

Local or special notices, five lines or less, will be inserted for $3 each insertion; over five lines, 50 cts. per line.

All advertising bills payable in advance, or at furthest, after the issue of the first number containing the advertisement. All transient advertisements to be paid for in advance.

☞CONTRIBUTIONS SOLICITED.

Exclusively Editorial Communications should be addressed to the Editor.

The latest number of the REGISTER may be ordered with or without other goods at any time, and all letters should be addressed to

**SAM'L A. CROCKER & CO., Ohio Dental and Surgical Depot, Cincinnati, O.**

